# Epicardial adipose tissue volume stratifies atrial fibrillation subtypes in reduced ejection fraction heart failure: a CT-based predictor

**DOI:** 10.3389/fcvm.2026.1780414

**Published:** 2026-04-01

**Authors:** Ji-Fang Ma, Qi-Fan Hua, You Zhou, Juan Hu, Wei-Feng Song, Ke Chen, Xiao-Biao Zang, Er-Peng Liang, Yong-Hui Zhao, Zhi-Ping Guo, Hai-Xia Fu

**Affiliations:** 1 Department of Cardiology, Central China Fuwai Hospital of Zhengzhou University, Fuwai Central China Cardiovascular Hospital, Zhengzhou, China; 2 Department of Radiology, Central China Fuwai Hospital of Zhengzhou University, Fuwai Central China Cardiovascular Hospital, Henan Provincial Key Laboratory of Cardiology Medical Imaging, Zhengzhou, China; 3 Henan Provincial Health Management Center, Fuwai Central China Cardiovascular Hospital, Zhengzhou, China

**Keywords:** atrial fibrillation, computed tomography, epicardial adipose tissue, heart failure, predictor

## Abstract

**Background:**

Epicardial adipose tissue (EAT) is involved in atrial fibrillation (AF) pathogenesis. Its specific role in stratifying AF subtypes among patients who also have reduced ejection fraction heart failure is not well defined.

**Objective:**

We aimed to compare EAT volume between AF patients with and without reduced ejection fraction heart failure, and to assess its link with AF subtypes.

**Methods:**

In this retrospective study, 224 AF patients (2018–2023) were classified into CAH group (combined AF and heart failure, LVEF < 50%, *n* = 90) and AF group (AF without heart failure, LVEF ≥50%, *n* = 134). Cardiac computed tomography (CT) was used to quantify EAT volume.

**Results:**

Overall EAT volume was similar between the CAH and AF groups (152.3 ± 61.6 vs. 166.8 ± 73.2 mL, *P* = 0.12). However, patients with persistent AF (PsAF) had significantly larger EAT volumes than those with paroxysmal AF (PaAF) in the entire cohort (166.9 ± 71.0 vs. 145.1 ± 61.0 mL, *P* = 0.04). This difference was driven entirely by the CAH subgroup, where PsAF patients had greater EAT than PaAF patients (160.2 ± 59.5 vs. 127.8 ± 13.4 mL, *P* = 0.03); no such difference existed in the AF subgroup. EAT volume correlated with left atrial (LA) diameter overall (*r* = 0.35, *P* < 0.05). While EAT volume predicted PsAF with high sensitivity but very low specificity in all patients (AUC = 0.60, sensitivity = 97.8%, specificity = 1.6%), its performance was more balanced within the CAH subgroup (AUC = 0.66, sensitivity = 86.8%, specificity = 45.5%). Notably, in this CAH group, LA diameter lost its predictive value for PsAF (AUC = 0.58, *P* = 0.28).

**Conclusion:**

In patients with heart failure, increased epicardial adipose tissue (EAT) volume is linked to the progression from paroxysmal to persistent atrial fibrillation (AF). Measuring EAT volume may therefore serve as a useful imaging biomarker to identify those at higher risk of AF progression, which could help guide more personalized management.

## Background

Atrial fibrillation (AF) is a frequent comorbidity in chronic heart failure (HF), with a reported prevalence of 10%–50% (1). Its pathogenesis involves atrial substrate remodeling (ASR) (2), a process in which inflammatory activation is a key contributor (3). Epicardial adipose tissue (EAT), a metabolically active endocrine depot that envelops approximately 80% of the heart's surface and accounts for up to 20% of its weight, has emerged as a significant player in this context. EAT secretes pro-inflammatory cytokines (e.g., IL-6, TNF-α) and adipokines (e.g., adiponectin) that can permeate the adjacent atrial myocardium, thereby promoting fibrosis and electrical heterogeneity (1, 4). Consequently, the expansion of EAT is closely linked to the progression of atrial fibrosis.

Elevated peri-atrial EAT volume is associated with an increased prevalence and severity of new-onset AF (5, 6). Notably, EAT volume has been identified as an independent predictor of AF occurrence, even after adjusting for conventional risk factors including obesity, dyslipidemia, and hypertension (5). In patients with non-valvular AF, EAT thickness correlates with distinct atrial conduction disturbances, such as prolonged P-wave duration, intra-atrial conduction block, and PR-interval prolongation (6).

The relationship between EAT and HF is complex and not fully elucidated. Kugler et al. (7) observed heightened inflammatory activity and increased macrophage infiltration within the EAT of AF patients with coexisting HF. Although EAT volume may be greater in HF patients compared to healthy individuals, advanced HF can paradoxically lead to systemic fat loss and reduced EAT volume (8). Moreover, EAT accumulation is associated with worse HF prognosis, demonstrating a strong unadjusted relationship with the composite endpoint of HF hospitalization and all-cause mortality (9, 10). Wang et al. (11) reported that patients with heart failure with preserved ejection fraction (HFpEF) had larger EAT volumes than those with heart failure or controls; however, their study did not analyze AF subtypes. Importantly, patients with persistent AF (PsAF) exhibit significantly greater EAT thickness and volume than those with paroxysmal AF (PaAF) (6), implying that EAT accumulation may actively drive AF progression in the setting of HF. Despite these insights, the specific association between EAT distribution and AF progression in the heart failure population remains underexplored.

To address this gap, our study used cardiac CT to systematically evaluate EAT volume in AF patients with and without heart failure. We focused on its association with AF subtypes and compared its predictive utility for identifying PsAF against that of conventional echocardiographic parameters like LA diameter.

## Methods

### Study population

We conducted a single-center, retrospective study of patients admitted for AF between 2018 and 2023. Included patients were aged ≥18 years with an AF diagnosis per contemporary guidelines (1). We excluded those with prior cardiac surgery or ablation, severe hepatic/renal dysfunction, malignancy, or active infection. Based on transthoracic echocardiography, patients were categorized into two groups: CAH group: combined AF and heart failure, with left ventricular ejection fraction (LVEF) <50% (*n* = 90); and AF group: AF with LVEF ≥ 50% (*n* = 134).

**Figure 1:**
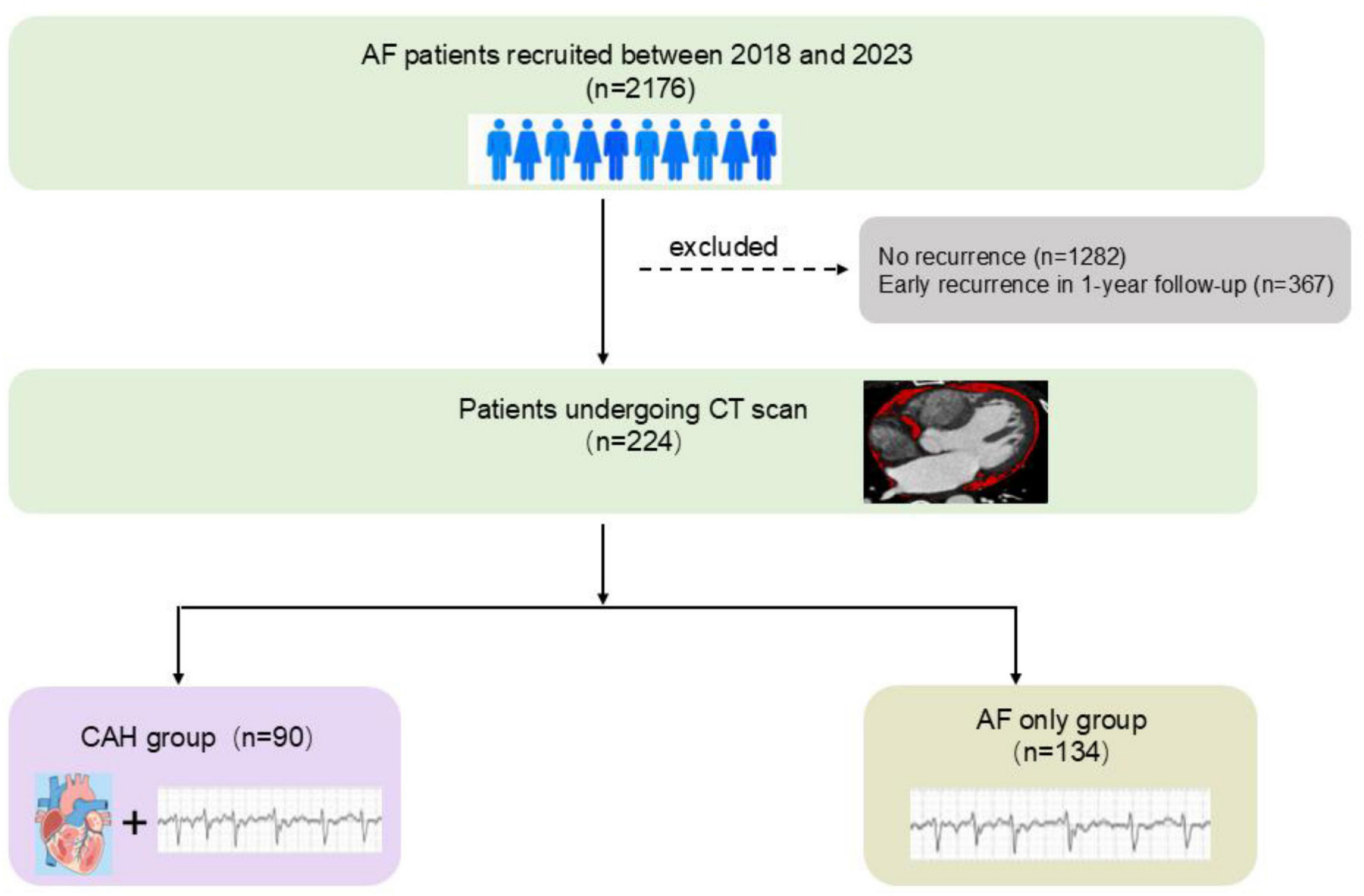
The protocol and flow of this study. PaAF, paroxysmal atrial fibrillation; VLR, very late recurrence; CAH, combined atrial fibrillation and reduced ejection fraction heart failure; AF, atrial fibrillation.

AF was classified as paroxysmal (PaAF, self-terminating episodes lasting ≤7 days) or persistent (PsAF, sustained episodes >7 days) (1). The study design is summarized in Figure 1.

### EAT quantification

**Figure 2:**
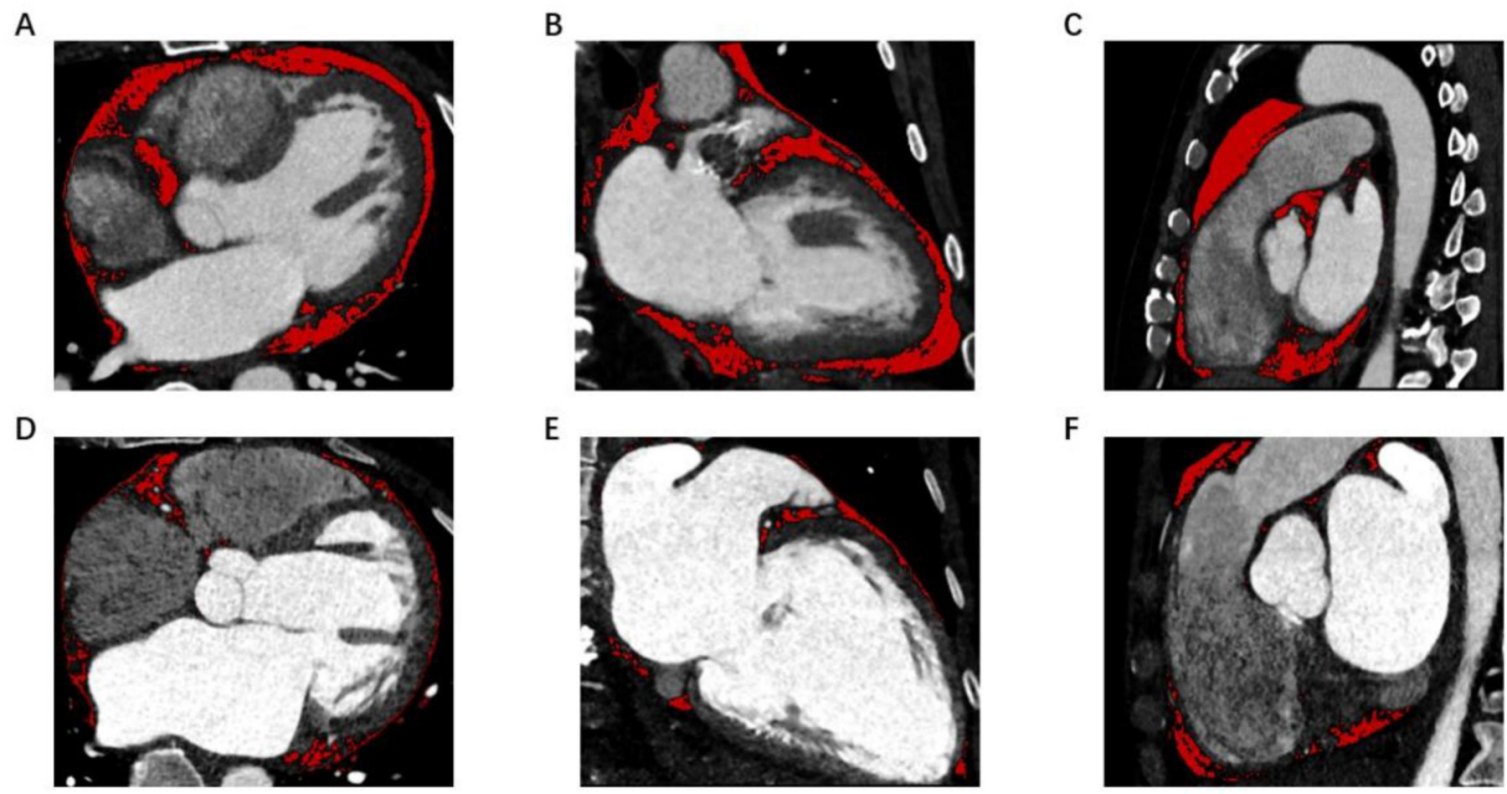
Measurement of EAT volume in different type of AF with heart failure. EAT volume was larger in PsAF **(A–C)** than PaAF **(D–F)** in patients with Heart Failure. The EAT volume was analyzed using CT image data.

All patients underwent coronary CT angiography within one month of AF diagnosis, using a prospective ECG-gated protocol. EAT volume was quantified offline using dedicated software (Siemens Healthineers, CT Cardiac Risk Assessment plugin). Adipose tissue was defined by voxels with attenuation between −50 and −200 Hounsfield Units within the pericardial sac, from the pulmonary artery bifurcation to the diaphragm (Figure 2).

### Data collection

We collected baseline demographics, comorbidities, and laboratory data including NT-proBNP. Echocardiographic measurements included LA diameter, left ventricular end-diastolic diameter (LVEDD), left ventricular end-systolic diameter (LVESD), and LVEF. Medication regimens were also recorded.

### Statistical analysis

Continuous data are presented as mean ± standard deviation and compared using Student's t-test or the Mann–Whitney *U* test, as appropriate. Categorical variables are shown as counts (percentages) and compared with the chi-square or Fisher's exact test. To evaluate whether the association between EAT volume and persistent AF was independent of heart failure severity, we performed a multivariate binary logistic regression analysis within the CAH cohort. The presence of persistent AF (vs. paroxysmal AF) was entered as the dependent variable. EAT volume was included as the primary independent variable, and NT-proBNP levels were entered as a covariate to adjust for potential confounding by the degree of hemodynamic stress. We used receiver operating characteristic (ROC) curve analysis to evaluate the performance of EAT volume in predicting PsAF. The correlation between EAT volume and LA diameter was assessed with Pearson's coefficient. A two-sided *P* value < 0.05 was considered statistically significant.

## Results

### Baseline characteristics

The study included 224 patients (72.3% male, mean age 61.0 ± 10.0 years). The CAH and AF groups were similar in age, sex, weight, smoking status, and alcohol use. However, diabetes (28.9% vs. 9.7%, *P* < 0.01) and coronary artery disease (41.1% vs. 25.4%, *P* = 0.01) were more common in the CAH group. As expected, the CAH group had significantly higher NT-proBNP, larger LVEDD and LVESD (all *P* < 0.01, Table 1).

**Table 1:** Baseline characteristics of the CAH and AF population.

Parameters	Total (*n* = 224)	CAH group (*n* = 90)	AF group (*n* = 134)	*P* value
Male (*n*, %)	162 (72.3)	62 (68.9)	100 (74.6)	0.35
Age (years)	61.0 ± 10.0	60.0 ± 9.0	62.0 ± 9.0	0.29
Body Weight (kg)	73.9 ± 13.7	72.4 ± 12.7	75.0 ± 14.3	0.16
BMI	25.3 ± 3.8	25.9 ± 3.8	24.9 ± 3.8	0.062
PsAF type (*n*, %)	163 (72.8)	68 (75.6)	95 (70.9)	0.44
Smoke (*n*, %)	68 (30.4)	27 (30)	41 (30.6)	0.90
Alcohol (*n*, %)	60 (26.8)	26 (28.9)	34 (25.4)	0.58
Hypertension (*n*, %)	97 (43.3)	32 (35.6)	65 (48.5)	0.04*
T2DM (*n*, %)	39 (17.4)	26 (28.9)	13 (9.7)	<0.01*
Coronary heart disease (*n*, %)	71 (31.7)	37 (41.1)	34 (25.4)	0.01*
Stroke (*n*, %)	27 (12.1)	8 (8.9)	19 (14.2)	0.23
NT-proBNP (pg/ml)	1,259.6 (465.8, 1,345.8)	1,802.3 (743.0, 2,028.2)	750.8 (275.8, 914.0)	<0.01*
Average CHA_2_DS_2_-VASc score	2.9 ± 1.4	2.9 ± 1.4	2.8 ± 1.5	0.90
LA (mm)	43.6 ± 6.0	44.0 ± 6.0	43.4 ± 6.0	0.44
LVEF (%)	52.3 ± 12.4	38.6 ± 6.1	61.6 ± 4.4	<0.01*
LVEDD (mm)	51.7 ± 7.8	57.0 ± 8.8	48.2 ± 4.3	<0.01*
LVESD (mm)	37.7 ± 9.1	46.1 ± 8.2	32.0 ± 3.4	<0.01*

CAH group, combined AF and Heart failure; AF group, atrial fibrillation without heart failure; PsAF, persistent atrial fibrillation; T2DM, type 2 diabetes mellitus; CHA_2_DS_2_-VASc, congestive heart failure, hypertension, age ≥75 years, diabetes mellitus, prior stroke, transient ischemic attack, or thromboembolism, vascular disease, age 65–74 years, sex category (female); LA, left atrium; LVEF, left ventricular ejection fraction; LVEDD, left ventricular end-diastolic dimension; LVESD, left ventricular end-systolic dimension. *P* value < 0.05 means statistically significant. Values are mean ± SD or % or median (25th, 75th).

*P value<0.05 means statistically significant.

### EAT volume analysis

**Figure 3:**
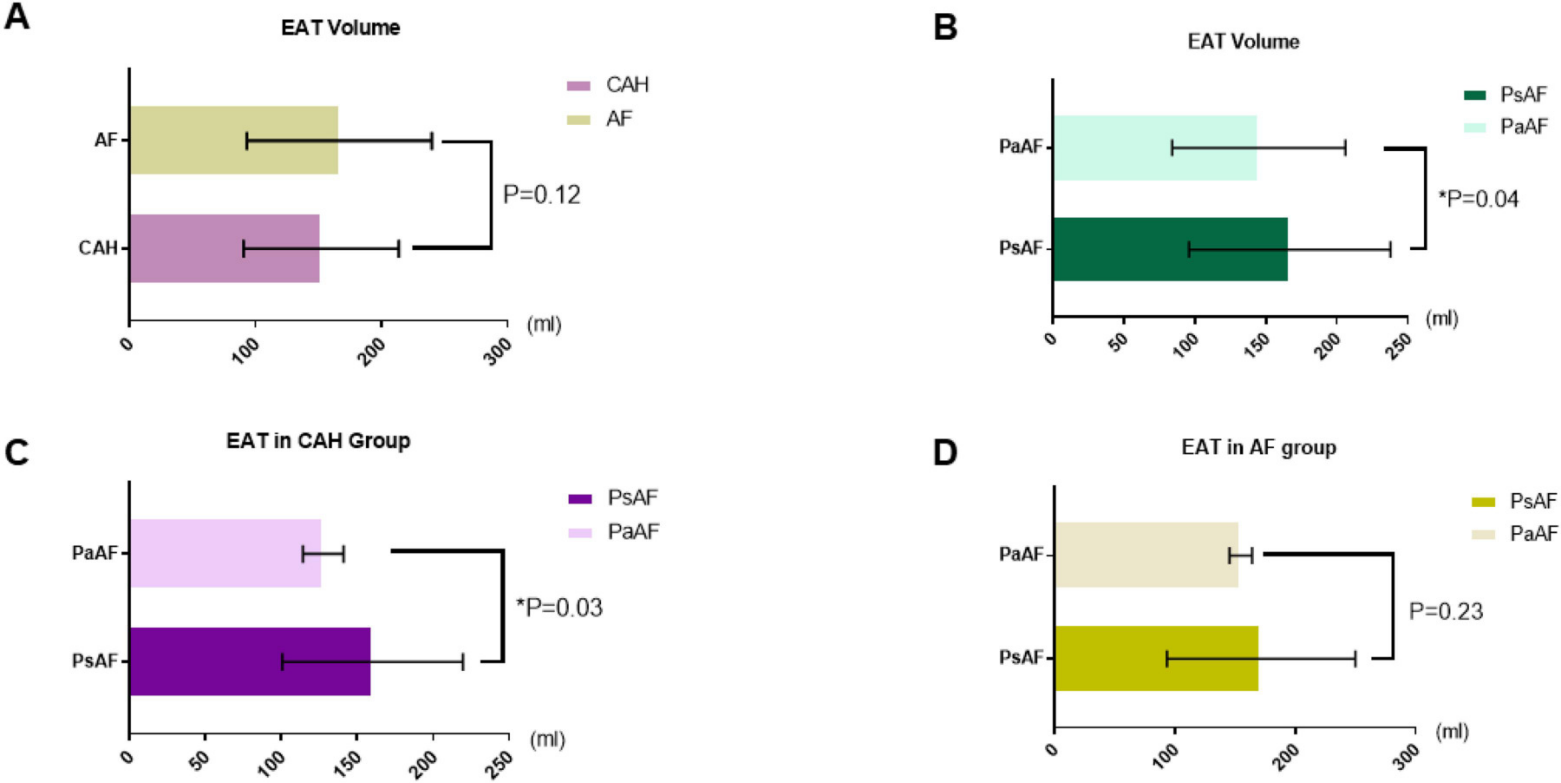
Comparative analysis of epicardial adipose tissue (EAT) volumes across atrial fibrillation (AF) subtypes. **(A)** Population-level analysis demonstrated comparable EAT volumes between CAH and AF cohorts (152.3 ± 61.6 mL vs. 166.8 ± 73.2 mL, *P* = 0.12); **(B)** In the total study population, persistent AF (PsAF) patients exhibited significantly higher EAT volumes than paroxysmal AF (PaAF) cases (166.9 ± 71.0 mL vs. 145.1 ± 61.0 mL, *P* = 0.04*); **(C,D)** Subgroup analysis revealed differential patterns: In the Combined Atrial fibrillation and Heart failure (CAH) cohort, PsAF patients showed substantially larger EAT volumes compared to PaAF (160.2 ± 59.5 mL vs. 127.8 ± 13.4 mL, *P* = 0.03*)**(C)**; In the AF cohort, no significant EAT volume disparity was observed between PaAF and PsAF subtypes (171.7 ± 78.2 mL vs. 154.9 ± 9.3 mL, *P* = 0.23) **(D)** *Data presented as mean ± SD. Statistical significance was determined using Student's *t*-test for group comparisons. *P* *<* *0.05 considered statistically significant*.

We found no significant difference in mean EAT volume between the CAH and AF groups (152.3 ± 61.6 vs. 166.8 ± 73.2 mL, *P* = 0.12, Figure 3A). However, when analyzed by AF subtype, patients with PsAF had larger EAT volumes than those with PaAF in the overall cohort (166.9 ± 71.0 vs. 145.1 ± 61.0 mL, *P* = 0.04, Figure 3B). Subgroup analysis revealed this difference was significant within the CAH cohort (PsAF: 160.2 ± 59.5 vs. PaAF: 127.8 ± 13.4 mL, *P* = 0.03, Figure 3C) but not within the AF only cohort (*P* = 0.23, Figure 3D). Multivariate logistic regression analysis within the CAH cohort confirmed that EAT volume remained independently associated with persistent AF after adjusting for NT-proBNP levels (*P* = 0.026, OR 1.12, 95% CI 1.10–1.13).

### Clinical features by AF subtype

Of the 224 patients, 163 had PsAF and 61 had PaAF. Compared to PaAF patients, those with PsAF were more often male, had higher body weight, reported more alcohol use, and had larger LA diameters (all *P* < 0.05). These differences were absent in the CAH subgroup but remained evident in the AF subgroup (Table 2).

**Table 2:** Clinical characteristics across different AF types.

Parameters	Total (*n* = 224)			CAH group (*n* = 90)			AF group (*n* = 134)		
	PsAF (*n* = 163)	PaAF (*n* = 61)	*P* value	PsAF (*n* = 68)	PaAF (*n* = 22)	*P* value	PsAF (*n* = 95)	PaAF (*n* = 39)	*P* value
Male (*n*, %)	128 (78.5)	34 (55.7)	<0.05*	48 (70.6)	14 (63.6)	0.54	80 (84.2)	20 (51.3)	<0.05*
Age (years)	60.8 ± 10.0	62.4 ± 10.6	0.28	60.6 ± 11.4	59.4 ± 13.5	0.68	60.9 ± 9.0	64.2 ± 8.3	0.05
Body Weight (kg)	75.1 ± 14.0	70.8 ± 12.5	0.04*	73.4 ± 12.4	69.0 ± 13.3	0.16	76.4 ± 15.0	71.8 ± 12.0	0.09
Smoke (*n*, %)	51 (31.5)	17 (27.9)	0.60	20 (29.4)	7 (31.8)	0.83	31 (33.0)	10 (25.6)	0.40
Alcohol (*n*, %)	51 (31.5)	9 (14.8)	0.01*	23 (33.8)	3 (13.6)	0.07	28 (29.8)	6 (15.4)	0.08
Hypertension (*n*, %)	72 (44.7)	25 (41)	0.62	24 (35.3)	8 (36.4)	0.93	48 (51.6)	17 (43.6)	0.40
T2DM (*n*, %)	25 (15.3)	14 (23.0)	0.18	18 (26.5)	8 (36.4)	0.37	7 (7.4)	6 (15.4)	0.20
CHD (*n*, %)	52 (31.9)	19 (31.1)	0.91	29 (42.6)	8 (36.4)	0.60	23 (24.2)	11 (28.2)	0.63
Stroke (*n*, %)	19 (11.7)	8 (13.1)	0.77	4 (5.9)	4 (18.2)	1.00	15 (15.8)	4 (10.3)	0.40
LA (mm)	44.6 ± 5.5	40.9 ± 6.3	<0.05*	44.5 ± 5.6	42.4 ± 6.8	0.16	44.7 ± 5.4	40.1 ± 6.0	<0.05*
LVEF (%)	51.6 ± 12.6	54.2 ± 11.8	0.17	38.3 ± 6.5	39.6 ± 5.0	0.38	61.3 ± 4.6	62.4 ± 3.8	0.83
LVEDD (mm)	52.2 ± 7.5	50.5 ± 8.5	0.15	57.2 ± 8.1	56.6 ± 11.0	0.78	48.6 ± 4.5	47.1 ± 3.7	0.06
LVESD (mm)	38.2 ± 8.9	36.3 ± 9.5	0.16	46.3 ± 7.7	45.7 ± 9.9	0.76	32.4 ± 3.5	31.0 ± 2.7	0.03*

Values are mean ± SD or % or median (25th,75th). EAT, epicardial adipose fat; PsAF, persistent atrial fibrillation; PaAF, paroxysmal atrial fibrillation; LA, left atrium; LVEF, left ventricular ejection fraction; LVEDD, left ventricular end-diastolic dimension; LVESD, left ventricular end-systolic dimension.

*P value<0.05 means statistically significant.

### Correlation between EAT volume and LA size

**Figure 4:**
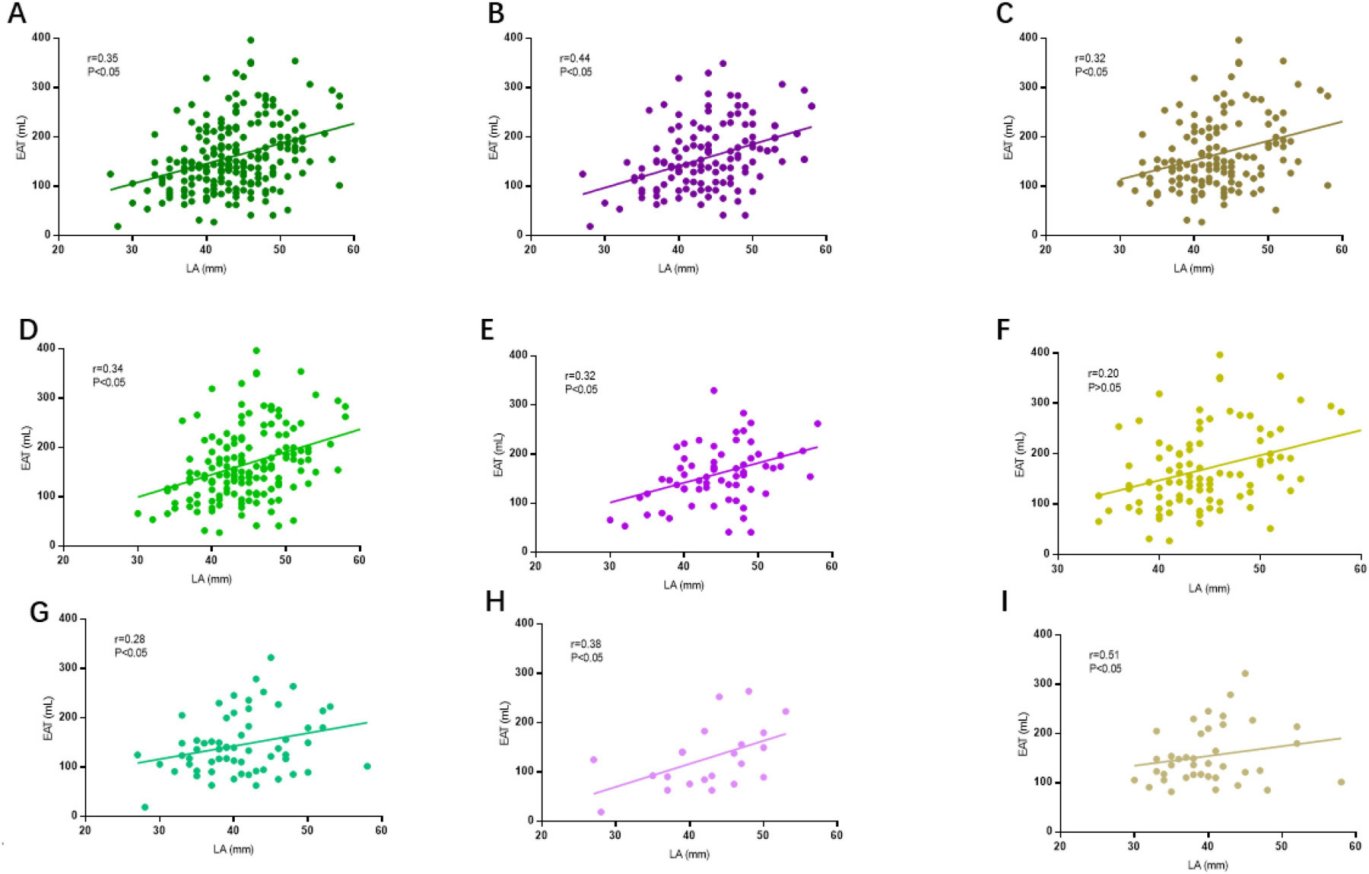
Association between left atrial (LA) volume and epicardial adipose tissue (EAT) volume stratified by AF subtype. **(A–C)** Overall AF population analysis: Significant positive correlations were observed between LA and EAT volumes across all cohorts: General AF population: *r* = 0.35, *P* < 0.05 **(A)**; Combined Atrial fibrillation and Heart failure (CAH) subgroup: *r* = 0.44, *P* < 0.05 **(B)**; AF subgroup: *r* = 0.32, *P* < 0.05 **(C,D–F)** Persistent AF (PsAF) subgroup analysis: Significant correlations persisted in the general population (*r* = 0.34, *P* < 0.05) **(D)**and CAH subgroup (*r* = 0.32, *P* < 0.05) **(E)**; No significant association was found in the AF subgroup (*r* = 0.20, *P* = 0.18) **(F)**; **(G–I)** Paroxysmal AF (PaAF) subgroup analysis: Significant correlations emerged in the general population (*r* = 0.28, *P* < 0.05) **(G)**, CAH subgroup (*r* = 0.38, *P* < 0.05) **(H)**, and AF subgroup (*r* = 0.51, *P* < 0.05) **(I)**. Pearson correlation coefficients with two-tailed significance testing. LA volume measured by 3D echocardiography; EAT volume quantified via cardiac CT using −30 to −190 Hounsfield unit thresholds.

EAT volume showed a positive correlation with LA diameter in the overall cohort (*r* = 0.35, *P* < 0.05), as well as in both the CAH (*r* = 0.44, *P* < 0.05) and AF (*r* = 0.32, *P* < 0.05) subgroups (Figures 4A–C). Stratifying by AF subtype, this correlation remained significant for PsAF patients in the overall and CAH groups, but not in the AF subgroup. Strong correlations were observed across all PaAF subgroups (Figures 4D–I).

### Predictive performance of EAT volume for PsAF

**Figure 5:**
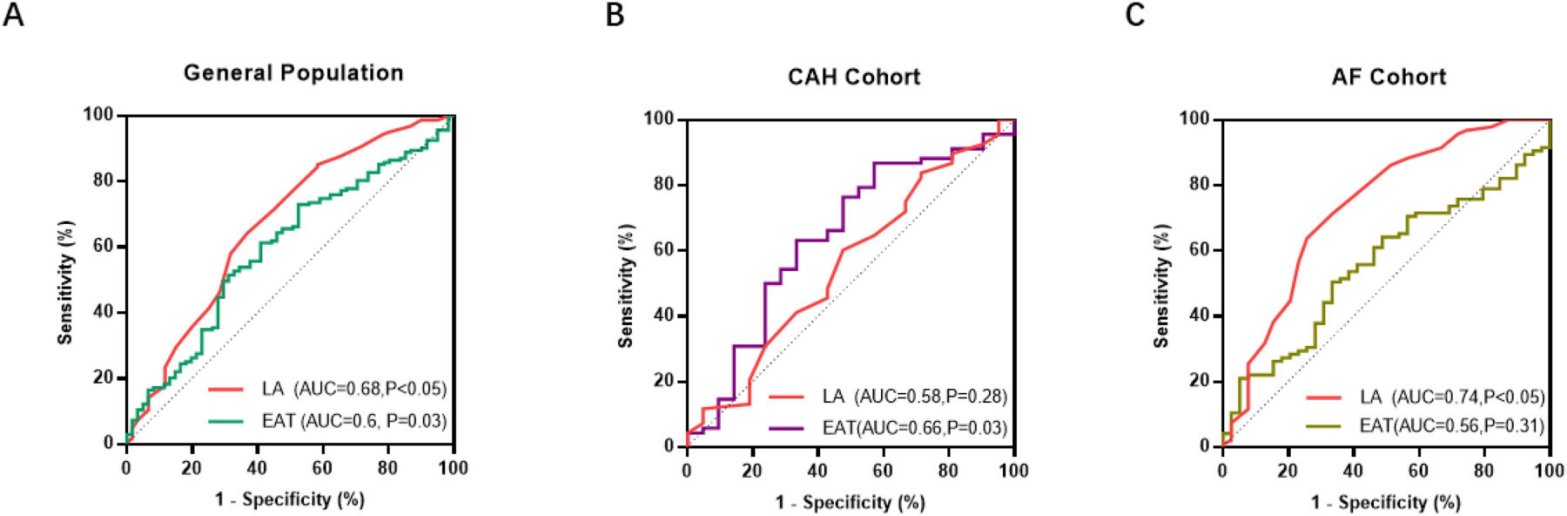
Diagnostic performance of left atrial diameter (LA) and epicardial adipose tissue (EAT) volume for persistent atrial fibrillation (PsAF) stratification. **(A)** Receiver operating characteristic (ROC) curve analysis in the general population demonstrated high sensitivity but low specificity of EAT volume for PsAF detection (sensitivity: 97.8%, specificity: 1.6%, AUC: 0.60.*P* = 0.03), as well as LA (AUC = 0.68,*P* < 0.05). **(B,C)** Subgroup stratification revealed heterogeneous diagnostic utility. In the Combined Atrial fibrillation and Heart failure (CAH) cohort, EAT volume showed excellent sensitivity and moderate specificity (sensitivity: 86.8%, specificity: 45.5%, AUC: 0.66, *P* = 0.03), the LA had weak specificity (AUC = 0.58, *P* = 0.28) **(B)** In the Atrial Fibrillation (AF) cohort, EAT volume exhibited improved specificity (sensitivity: 50.5%, specificity: 66.7%, AUC: 0.56, *P* = 0.31), but LA demonstrated improved specificity (AUC = 0.74, *P* < 0.05) **(C)** *Data presented as point estimates with 95% confidence intervals. AUC interpretation: 0.5 = no discrimination; 0.7−0.8 = acceptable; 0.8−0.9 = excellent. *P* *<* *0.05 vs. AF cohort by DeLong's test*.

ROC analysis showed that in the overall cohort, EAT volume predicted PsAF with very high sensitivity (97.8%) but negligible specificity (1.6%), resulting in an AUC of 0.60 (*P* = 0.03). LA diameter was a better predictor overall (AUC = 0.68, *P* < 0.05, Figure 5A). The pattern differed markedly in subgroups. Within the CAH cohort, EAT volume demonstrated a more balanced predictive profile (AUC = 0.66, sensitivity = 86.8%, specificity = 45.5%, *P* = 0.03), while LA diameter was not predictive (AUC = 0.58, *P* = 0.28, Figure 5B). Conversely, in the AF cohort, EAT volume had poor predictive value (AUC = 0.56, *P* = 0.31), and LA diameter was a strong predictor (AUC = 0.74, *P* < 0.05, Figure 5C).

## Discussion

**Figure 6:**
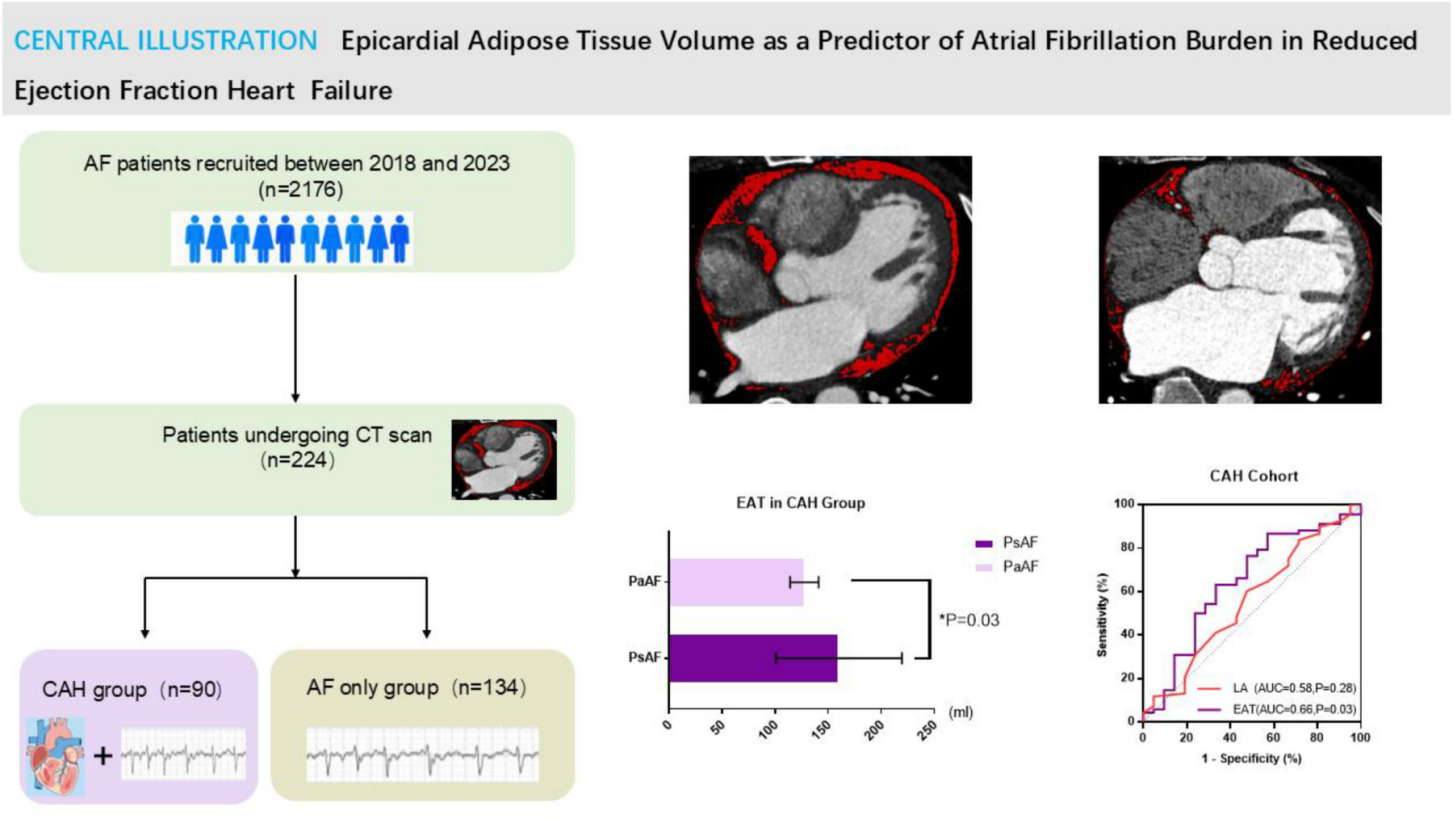
Epicardial adipose tissue volume as a predictor of atrial fibrillation burden in reduced ejection fraction heart failure. Left pane: The protocol and flow of this study. Right superior pane: The EAT volume was mraked by red areas via CT scan. PsAF patient(Left) had larger EAT than PaAF (right) in patients with heart failure. Right inferior pane: Left: In CAH group, EAT was significantly larger in PsAF than PaAF; Right: In CAH cohort, EAT volume showed excellent sensitivity and moderate specificity (sensitivity: 86.8%, specificity: 45.5%, AUC: 0.66, *P* = 0.03), the LA had weak specificity (AUC = 0.58, *P* = 0.28).

This study provides a focused investigation into the association between epicardial adipose tissue (EAT) volume and atrial fibrillation (AF) subtypes, with particular emphasis on the context of reduced ejection fraction heart failure. The principal finding is that increased EAT volume is significantly associated with persistent AF (PsAF) compared to paroxysmal AF (PaAF) within the heart failure population. More importantly, our analysis reveals a divergent predictive utility: in patients with reduced ejection fraction heart failure, EAT volume demonstrates a significant, albeit modest, ability to identify PsAF, whereas the conventional echocardiographic marker—left atrial (LA) diameter—fails to do so. This pattern stands in contrast to AF patients without HF, in whom LA diameter remains a robust predictor (Figure 6). These observations suggest that the pathophysiological drivers and clinically relevant biomarkers of AF progression may differ substantially depending on the presence and type of comorbid heart failure.

The strong link between obesity and both AF incidence and post-ablation recurrence is well established (1, 12). EAT volume, as assessed by CT or MRI, has been independently associated with AF occurrence and recurrence (13, 14). Notably, data from the Framingham Heart Study indicated that this association remains significant even after adjustment for traditional risk factors (5). Elevated EAT volumes have been observed in patients with both paroxysmal and persistent AF when compared to individuals in sinus rhythm (15), supporting the role of EAT volume as an independent risk factor for the development and maintenance of AF.

The pro-arrhythmic mechanisms likely involve EAT-mediated atrial fibrosis (15–17). EAT may directly infiltrate the atrial myocardium (15) and facilitate cellular crosstalk (16). As an active endocrine organ, EAT secretes cytokines that act on cardiomyocytes through paracrine, vasocrine, or systemic pathways (17), thereby promoting fibrosis and electrical instability (18). EAT size correlates significantly with atrial conduction delays (6), and its accumulation contributes to heterogeneous atrial voltage and conduction abnormalities (19). This creates a stable substrate for AF maintenance. Our finding of larger EAT volumes in patients with PsAF vs. PaAF aligns with this mechanistic framework and corroborates prior studies linking EAT to AF persistence. However, our study extends this knowledge by demonstrating that this association is not uniform across all AF patients but is particularly salient in the presence of reduced ejection fraction heart failure.

Given the established role of EAT in promoting fibrosis, along with the recognized association between left atrial (LA) size and fibrosis (20), EAT volume is significantly correlated with LA enlargement and dysfunction (20). Persistent AF is characterized by more pronounced atrial structural remodeling—including LA enlargement and fibrosis—compared with paroxysmal AF (21). In line with this, our study confirmed significantly larger EAT volumes in patients with PsAF vs. those with paroxysmal AF in the overall cohort. Moreover, the positive correlation observed between EAT volume and LA size across all participants supports a meaningful impact of EAT on atrial structural remodeling.

Previous studies using canine HF models have demonstrated that tissue remodeling is more prominent in the atria than in the ventricles (22), with AF occurrence consistently linked to the development of atrial substrate remodeling (22–24). Reduced LVEF is associated with structural remodeling of both the atria and ventricles (25, 26). Furthermore, in the setting of HF, AF is associated with a relatively modest increase in atrial size but a substantially greater increase in atrial fibrosis (22). Our correlation analyses further support the link between EAT and structural remodeling. The positive correlation between EAT volume and LA diameter across the cohort reinforces the interplay between adipose tissue and chamber dilation. The particularly strong correlation observed in the PaAF subgroups, especially within the non-HF cohort, might suggest that in earlier AF stages or in the absence of advanced heart failure, EAT accumulation and atrial enlargement progress in a more coupled manner. The attenuation of this correlation in some PsAF subgroups could reflect the emergence of other dominant remodeling factors in persistent disease.

Although the CAH and AF groups were comparable in terms of AF subtype distribution (*P* = 0.44), we acknowledge that the significantly higher NT-proBNP levels in the CAH group may reflect a greater degree of hemodynamic stress and neurohormonal activation in these patients. This raises the possibility that the more persistent nature of AF observed in the CAH subgroup could be partially influenced by the severity of heart failure decompensation rather than solely by EAT volume. However, several observations suggest that this potential bias does not fully account for our findings. First, within the CAH group, all patients met the criteria for reduced ejection fraction heart failure (LVEF < 50%), and no significant differences in clinical signs of heart failure decompensation (e.g., NYHA class, peripheral edema, or jugular venous distension) were noted between those with PsAF and PaAF. Second, we performed a multivariate logistic regression analysis within the CAH cohort, adjusting for NT-proBNP levels. The results showed that EAT volume remained a significant predictor of persistent AF even after adjustment for NT-proBNP (OR = 1.12, 95% CI: 1.10–1.13, *P* = 0.026), suggesting that the association between EAT and AF persistence is at least partially independent of the degree of hemodynamic stress reflected by NT-proBNP. Third, the divergent predictive patterns—where EAT volume predicted PsAF in the CAH group but not in the AF group—suggest a context-dependent role for EAT that extends beyond simple markers of heart failure severity. Nonetheless, we acknowledge that residual confounding by heart failure severity cannot be completely excluded, and future prospective studies with comprehensive assessments of clinical decompensation are needed to validate these findings.

We postulate that EAT acts as a potent local amplifier of these deleterious processes within the immediate pericardiac environment. In heart failure, the atrial myocardium is already primed for remodeling; the additional burden of inflammatory and fibrotic signals from adjacent EAT may critically accelerate the development of a persistent AF substrate. Consequently, EAT volume in this context may serve as a more sensitive indicator of the overall “fibrotic/inflammatory burden” specific to the atria than gross anatomical changes reflected by LA diameter. Conversely, in patients without the profound systemic remodeling of heart failure, atrial enlargement (LA diameter) may remain a more direct and sufficient morphological correlate of AF-related atrial remodeling, explaining its sustained predictive power in the AF only group.

This underscores a crucial point: the clinical relevance of EAT is not merely a function of its quantity but of its context-dependent pathological activity. Our study shifts the focus from comparing EAT volume between disease states to evaluating its predictive role within a specific, high-risk state (heart failure). Even a “moderate” amount of EAT may exert a disproportionately large arrhythmogenic effect in the vulnerable atrial substrate of a failing heart, making it a valuable discriminatory biomarker within that population.

### Clinical implications and future directions

These findings carry practical implications for risk stratification. In heart failure patients with AF, assessment of EAT volume on routinely obtained cardiac CT scans could provide incremental prognostic information. Identifying those with higher EAT volumes might help clinicians pinpoint individuals at elevated risk for progressing to persistent AF, a state associated with worse outcomes and greater management complexity. This could inform decisions regarding the intensity of follow-up, the appropriateness of early rhythm control strategies, or the selection of patients for trials investigating novel therapies targeting adipose tissue biology, inflammation, or fibrosis.

Future research should aim to validate these findings in larger, prospective, multi-center cohorts. Longitudinal studies are essential to establish whether EAT volume progression temporally precedes and predicts AF subtype transition in heart failure. Incorporating more detailed characterization of EAT, such as its attenuation (a potential marker of inflammation) or spatial distribution around specific atrial regions, could refine its predictive value. Furthermore, exploring the interplay between EAT, circulating biomarkers of fibrosis and inflammation, and atrial tissue characteristics (e.g., via late gadolinium enhancement on MRI) would deepen the mechanistic understanding.

## Limitations

Our study has several limitations to consider. First, the retrospective, single-center design and the relatively small size of certain subgroups—particularly the persistent AF (PaAF) patients within the reduce ejection fraction heart failure cohort—may limit the generalizability of our findings and reduce the precision of these specific sub-analyses. Second, because this was a cross-sectional analysis, we cannot assess how EAT volume changes over time or establish any causal relationship with AF progression. While we followed standardized protocols for EAT measurement, technical variations in CT scanning could still influence the results. Finally, our findings apply specifically to heart failure with reduced ejection fraction (LVEF <50%) and may not extend to heart failure with preserved ejection fraction (HFpEF). These limitations highlight the need for cautious interpretation and support further validation in larger, prospective, and multi-center studies.

## Conclusion

EAT volume is associated with the progression from paroxysmal to persistent AF, and this relationship is especially pronounced in patients with heart failure. Importantly, within this high-risk population, EAT volume emerges as a predictor of persistent AF while LA diameter does not, highlighting its potential as a context-specific imaging biomarker. These findings support further investigation into the role of EAT in risk stratification and its potential as a therapeutic target in patients with concomitant AF and heart failure.

## Data Availability

The raw data supporting the conclusions of this article will be made available by the authors, without undue reservation.
